# Porphyrin Excretion Resulting From Mutation of a Gene Encoding a Class I Fructose 1,6-Bisphosphate Aldolase in *Rhodobacter capsulatus*

**DOI:** 10.3389/fmicb.2019.00301

**Published:** 2019-02-22

**Authors:** Hao Ding, Rafael G. Saer, J. Thomas Beatty

**Affiliations:** ^1^Department of Microbiology and Immunology, The University of British Columbia, Vancouver, BC, Canada; ^2^Department of Biology, Washington University in St. Louis, St. Louis, MO, United States; ^3^Department of Chemistry, Washington University in St. Louis, St. Louis, MO, United States

**Keywords:** porphyrin excretion, coproporphyrinogen III, HemN, fructose 1,6-bisphosphate aldolase, class I FBA, moonlight activity

## Abstract

This paper describes a mutant (called SB1707) of the *Rhodobacter capsulatus* wild type strain SB1003 in which a transposon-disrupted *rcc01707* gene resulted in a ∼25-fold increase in the accumulation of coproporphyrin III in the medium of phototrophic (anaerobic) cultures grown in a yeast extract/peptone medium. There was little or no stimulation of pigment accumulation in aerobic cultures. Therefore, this effect of *rcc01707* mutation appears to be specific for the anaerobic coproporphyrinogen III oxidase HemN as opposed to the aerobic enzyme HemF. The protein encoded by *rcc01707* is homologous to Class I fructose 1,6-bisphosphate aldolases, which catalyze a glycolytic reaction that converts fructose 1, 6-bisphosphate to dihydroxyacetone phosphate and glyceraldehyde 3-phosphate, precursors of pyruvate. There were significant differences in coproporphyrin III accumulation using defined media with individual organic acids and sugars as the sole carbon source: pyruvate, succinate and glutamate stimulated accumulation the most, whereas glucose suppressed coproporphyrin III accumulation to 10% of that of succinate. However, although quantitatively lesser, similar effects of carbon source on the amount of accumulated pigment in the culture medium were seen in a wild type control. Therefore, this mutation appears to exaggerate effects also seen in the wild type strain. It is possible that mutation of *rcc01707* causes a metabolic bottleneck or imbalance that was not rectified during growth on the several carbon sources tested. However, we speculate that, analogous to other fructose 1,6-bisphosphate aldolases, the *rcc01707* gene product has a “moonlighting” activity that in this case is needed for the maximal expression of the *hemN* gene. Indeed, it was found that the *rcc01707* gene is needed for maximal expression of a *hemN* promoter-*lacZ* reporter. With the decrease in *hemN* expression due to the absence of the *rcc01707* gene product, coproporphyrinogen III accumulates and is released from the cell, yielding the spontaneous oxidation product coproporphyrin III.

## Introduction

Cyclic tetrapyrroles such as hemes, chlorophylls, cobalamins and siroheme function primarily in electron transfer reactions. The anoxygenic photosynthetic bacteria such as *Rhodobacter capsulatus* are among the most-studied organisms for tetrapyrrole synthesis, in part due to a versatile metabolic capability, allowing for growth under dark aerobic, dark anaerobic, and illuminated anaerobic (phototrophic) conditions. Under aerobic conditions relatively small amounts of porphyrins are needed, mainly for hemes in cytochromes, whereas under phototrophic conditions much larger amounts are needed to provide bacteriochlorophyll (BChl) in addition to heme. *R. capsulatus* also synthesizes lesser amounts of cobalamin (vitamin B_12_).

**FIGURE 1:**
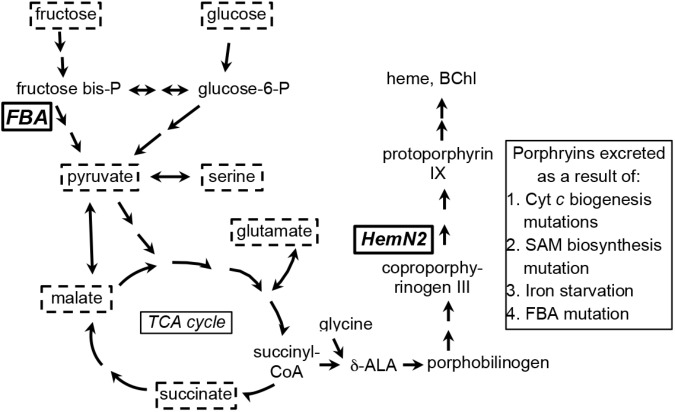
Simplified representation of porphyrin tetrapyrrole biosynthesis and metabolic pathways in *Rhodobacter capsulatus* relevant to the carbon substrates (enclosed in dashed boxes) used for growth of cultures in this work. Fructose is taken up by a phosphotransferase system, enters the cell as fructose-1-phosphate, and is catabolized to pyruvate through an Embden-Meyerhof-Parnas series of reactions (*FBA*, fructose bisphosphate aldolase). Glucose appears to be phosphorylated by glucokinase after entry into the cell, and glucose-6-phosphate is catabolized to pyruvate through an Entner–Doudoroff pathway. Fructose bisphosphate and glucose-6-phosphate can be interconverted through the sequential action of fructose 1–6 diphosphatase and phosphoglucose isomerase. Relationships between organic and amino acids are as shown. *HemN2*, the anaerobic coproporphyrinogen III oxidase encoded by *rcc02494*. The number of arrows in pathways may not necessarily correspond to the number of enzymatic reactions separating the substances shown (Eidels and Preiss, 1970; Conrad and Schlegel, 1977; Beatty and Gest, 1981a,b; Daniels et al., 1988; Biel and Biel, 1990; Wu and Saier, 1990; Goldman et al., 1997; Deshmukh et al., 2000; Dailey et al., 2017).

A review of *R. capsulatus* tetrapyrrole biosynthesis and its regulation was published by Zappa et al. (2010), and a condensed representation of the pathway in relation to central metabolism is shown in Figure 1. Briefly, the tetrapyrrole precursor δ-aminolevulinic acid (δ-ALA) is synthesized by the condensation of glycine and succinyl-CoA, catalyzed by δ-ALA synthase (HemA). In a series of reactions δ-ALA is converted to the tetrapyrrole uroporphyrinogen III. Three pathways branch from uroporphyrinogen III; the porphyrin branch (leading to heme and BChl), the siroheme branch, and the corrin branch (leading to cobalamin). In the porphyrin branch, uroporphyrinogen III is converted to coproporphyrinogen III by uroporphyrionogen III decarboxylase (HemE, also called CgdC), and coproporphyrinogen III is converted to protoporphyrinogen IX by either of two coproporphyrinogen III oxidases: HemN (also called HemZ and CgdH) under anaerobic conditions, or HemF under aerobic conditions. We herein use *hemN* to designate the gene (and HemN the Fe/S radical SAM enzyme) needed to convert coproporphyrinogen III to protoporphyrinogen IX under anaerobic conditions. Protoporphyrinogen IX is converted by protoporphyrinogen IX oxidase to protoporphyrin IX, the precursor in common to both heme- and BChl-specific pathways (Zappa et al., 2010; Dailey et al., 2017).

Several bacterial species contain more than one *hemN* gene, and the *R. capsulatus* genome was originally annotated in 2014 to contain three homologs, *hemN1* (*rcc00151*), *hemN2* (*rcc02494*), and *hemN3* (*rcc03489*). Although the exact function of each of these orthologs has not been rigorously studied, it appears that *hemN2* encodes the genuine anaerobic coproporphyrinogen III oxidase. There are several reasons for this conclusion. Firstly, *hemN1* and *hemN2*, but not *hemN3*, are induced in phototrophically grown (anaerobic) cells, and so *hemN3* appears to encode an aerobic enzyme (Kumka et al., 2017). Secondly, the *R. capsulatus* HemN1 and the *Escherichia coli* anaerobic heme degradation enzyme ChuW are 41% identical in a BLAST alignment, and the *R. capsulatus hemN1* is in an operon with a putative *chuX*, just as the *E. coli chuW* (LaMattina et al., 2016). In fact, the 2017 revision of the *R. capsulatus* genome annotation (NC_014034) describes *rcc00151* as a heme anaerobic degradation enzyme. Thirdly, In unpublished work kindly communicated by S. Zappa, it was found that deleting *hemN1* and *hemN3* did not change the growth or pigmentation phenotype, whereas attempts to delete *hemN2* failed. In other experiments on iron limitation these three genes responded differently, indicating different functions in iron homeostasis. These conclusions are supported by a tree based on a multiple sequence alignment of the three *R. capsulatus* proteins and the *E. coli* experimentally determined aerobic and anaerobic coproporphyrinogen III oxidases HemN and HemF, and the ChuW anaerobic heme degradation enzyme (Supplementary Figure S1).

Tetrapyrrole biosynthesis in *R. capsulatus* is regulated at the level of transcription and post-transcriptionally. A variety of global regulators including RegA-RegB, CrtJ, FnrL, AerR, HrBL and Irr control *hem* gene transcription. Three branches lead to the end products cobalamin, heme and BChl, which feed back into complex regulatory circuits and affect the production of each other (Zappa et al., 2010).

Under certain conditions (notably iron starvation) wild type (WT) *R. capsulatus* excretes coproporphyrinogen III (Cooper, 1963), and the extracellular accumulation of porphyrins by mutant *Rhodobacter* species has been documented (Lascelles, 1956, 1966; Biel and Biel, 1990; Sabaty et al., 2010). All of these mutants accumulate mainly coproporphyrin III, a spontaneous oxidation product of the intermediate coproporphyrinogen III. One class of porphyrin excretion mutants is defective in cytochromes *c*, and therefore incapable of photosynthetic growth (Biel and Biel, 1990; Goldman et al., 1997; Deshmukh et al., 2000). These mutants excrete a combination of coproporphyrin III and protoporphyrin IX only during growth under low oxygen concentrations. A *Rhodobacter sphaeroides* “PORF” mutant excreted large quantities of coproporphrin III, and had a mutation in the *metK* gene leading to a 70% decrease in intracellular S-adenosymethinine (SAM) content; therefore, it was proposed that the activity of SAM-dependent enzymes including the anaerobic coproporphyrinogen oxidase was inhibited, resulting in the accumulation of the metabolite coproporphrinogen III (Sabaty et al., 2010). Similarly, another study demonstrated that in a *copA* mutant of the purple bacterium *Rubrivivax gelatinosus*, which is hypersensitive to copper, the copper toxicity came from inhibition of radical SAM enzymes, including the anaerobic coproporphyrinogen oxidase (Azzouzi et al., 2013). In both studies, the accumulation of coproporphyrin III was observed only under microaerobic and anaerobic conditions, which was attributed to a decrease in the activity of the anaerobic coproporphyrinogen oxidase HemN. Therefore the reaction catalyzed by HemN enzymes appears to be a key step in the pathway that is affected by several factors in several species.

In this study, we identified a transposon-generated *R. capsulatus* mutant that excreted copious amounts of red pigment into the culture supernatant when grown under anaerobic, illuminated conditions. After purification by HPLC, mass spectrometry showed that the major component of the excreted red pigment appears to be coproporphyrin III, the oxidation product of coproporphyrinogen III. It was found that disruption of the *rcc01707* gene, encoding a Class I fructose 1,6-bisphosphate aldolase (FBA), caused the accumulation of coproporphyrin III. However compared to the difference between the WT and mutant strains, there were relatively minor effects on porphyrin accumulation when mutant cultures were grown on either sugar precursors of fructose 1,6-bisphosphate, or organic acids including pyruvate, which is a central metabolite derived from the aldolase activity of FBA on fructose 1,6-bisphosphate. Using a plasmid-borne *lacZ* reporter, we found that the expression of *hemN* was lower in the mutant strain SB1707 than in the WT strain SB1003. We speculate that this FBA, by analogy to other FBAs (Ritterson Lew and Tolan, 2013; Shams et al., 2014; Ziveri et al., 2017; Snaebjornsson and Schulze, 2018), has a “moonlighting” function in addition to its catalytic activity as an aldolase. This function would affect the anaerobic conversion of coproporphyrinogen III to protoporphyrin IX catalyzed by HemN, at least in part by stimulating expression of the *hemN* gene.

## Results

### Mutant SBT4-A13 Excretes a Red Pigment

**FIGURE 2:**
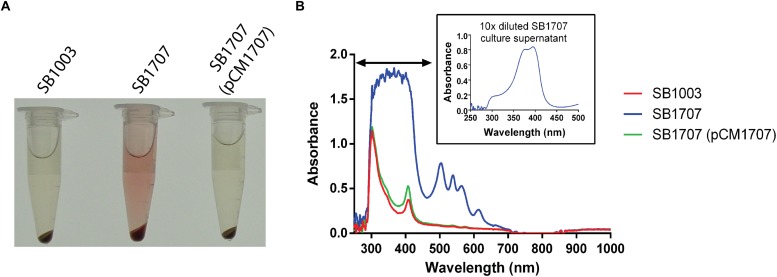
Accumulation of an extracellular red pigment in *R. capsulatus* cultures. The WT SB1003, mutant SB1707, and complemented mutant SB1707(pCM1707) strains were grown phototrophically in the complex medium YPS, and culture samples centrifuged to pellet cells. **(A)**, Pigmentation of culture supernatants. **(B)**, Absorbance spectra of culture supernatants; inset shows a dilution of the sample to reveal features in the Soret region (indicated by the horizontal double arrowhead line) that were off-scale in the undiluted sample of the mutant SB1707 culture. The peaks are located at around 400 (Soret band), 501, 535, 565, and 620 nm.

While screening a transposon library of *R. capsulatus* strain SBpG for mutants that exhibited elevated mCherry fluorescence (emission max = 610 nm), a mutant SBT4-A13 was found to accumulate a red-brown pigment in the culture medium when grown phototrophically (anaerobically with illumination) in the yeast extract/peptone complex medium YPS (Figure 2A). As shown in Figure 2B, the absorption spectrum of SBT4-A13 culture supernatant showed a peak pattern characteristic of a porphyrin, with strong absorption at around 400 nm (Soret band), and four peaks at around 501, 535, 565, and 620 nm (Cooper, 1963).

### Identification of the Excreted Pigment

**FIGURE 3:**
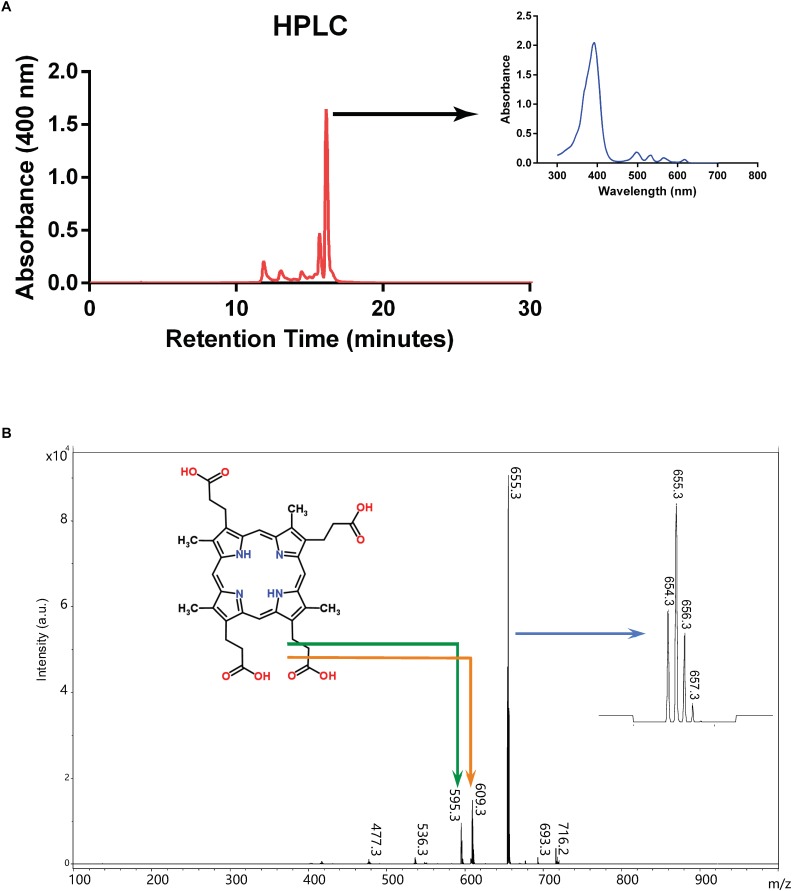
Purification and characterization of excreted porphyrin. **(A)** HPLC chromatogram from culture supernatant of mutant strain SB1707. Arrow indicates the absorption spectrum of the major peak. **(B)** Mass spectrum of the major peak from HPLC elution. The structure of coproporphyrin III is given (monoisotopic mass 658.3 Da when protonated as shown; http://www.chemspider.com/Chemical-Structure.315.html), with the bent arrows indicating peaks likely to have resulted from fragmentation as shown. The arrow pointing to the inset on the right indicates the expanded region around m/z 655.3 showing the isotopic distribution (see footnote 1).

We first tried to isolate and concentrate the pigment from culture supernatant by organic extraction into ethyl ether as previously described (Rebeiz, 2002), however the pigment was retained in the aqueous phase, indicating that the compound is hydrophilic. We therefore introduced filtered culture supernatant directly into HPLC, which revealed that a substance yielding a major peak absorbing at 400 nm had a retention time between 16 and 17 min (Figure 3A). This fraction was collected and analyzed by MALTI-TOF mass spectrometry (Figure 3B). The main peak had a [M+H]/*z* value of 655.3, the molecular weight of coproporphyrin III, and smaller peaks are consistent with its isotopic distribution^1^. These findings are also consistent with the fact that coproporphyrin III is hydrophilic because of four carboxylic acid groups (see Figure 3B), which explains the persistence of the pigment in the aqueous phase during organic solvent extraction.

Taken together, these data indicate that the main compound present in the culture supernatant of strain SBT4-A13 is coproporphyrin III (Gouda et al., 2012), the spontaneously oxidized product of the tetrapyrrole synthesis intermediate coproporphyrinogen III. The fluorescence emission of coproporphyrin peaks at 610 nm (Polo et al., 1988), similar to that of the mCherry fluorescent protein, which explains why this mutant was picked up in a screen for enhanced mCherry fluorescence.

### Disruption of ORF *rcc01707* Leads to a Coproporphyrin III-Accumulation Phenotype

The DNA sequence of the Tn5 insertion site in mutant strain SBT4-A13 revealed it to be located within codon 72 of the ORF *rcc01707*, encoding a 295 amino acid putative Class I FBA. To confirm that it was the disruption of *rcc01707* that caused the coproporphyrin III-accumulation phenotype, we transduced the kanamycin resistance (Km^R^) marker within *rcc01707* of mutant strain SBT4-A13 into the WT strain SB1003 using the gene transfer agent RcGTA. All of the Km^R^ transductants accumulated a red pigment in the culture medium when grown phototrophically in the complex medium YPS, with the same absorption spectrum as in cultures of the original SBT4-A13 mutant, and PCR amplification confirmed that these transductants contained a disrupted *rcc01707* gene, with an increased size corresponding to the insertion of the ∼1.8 kb Tn5. Introduction of a plasmid carrying *rcc01707* with its native promoter region and lacking flanking genes (pCM1707) into mutant strain SB1707 abolished the coproporphyrin III-accumulation phenotype (Figure 2).

### Phototrophic Growth and BChl Content Are Negatively Impacted in the Mutant Strain SB1707

Because large amounts of coproporphyrin are excreted by SB1707, we hypothesized that this decreases the synthesis of BChl, thus impairing phototrophic growth. It was found that the aerobic growth of mutant SB1707 was identical to that of the WT SB1003 in YPS medium (Supplementary Figure S2). However, SB1707 exhibited a longer lag phase and slower growth than strain SB1003 when transferred from aerobic dark to anaerobic phototrophic growth conditions (Supplementary Figure S3).

**FIGURE 4:**
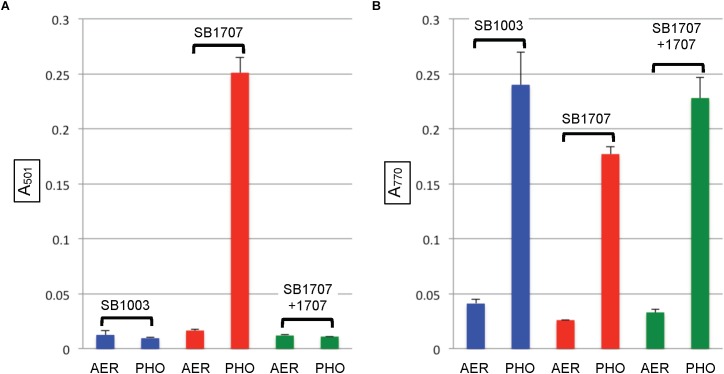
Pigment accumulation in cultures of the WT strain SB1003, mutant SB1707, and the mutant complemented with plasmid pCM1707, grown to the stationary phase in complex YPS medium aerobically (AER) or phototrophically (PHO). **(A)** Extracellular coproporphyrin III measured as A_501_, normalized to culture turbidity. **(B)** Intracellular bacteriochlorophyll *a* measured as A_770_, normalized to culture turbidity. Error bars give the range (*n* = 2).

When the mutant strain SB1707 was grown aerobically, the amount of coproporphyrin III accumulated (estimated by the A_501_ of culture supernatants) was essentially the same as in the WT strain SB1003 (Figure 4A). Because HemF converts coproporphyrinogen III to protoporphyrin IX under aerobic conditions, whereas HemN performs this reaction under anaerobic conditions (Dailey et al., 2017), it appears that the production or activity of HemN is affected by the mutation of *rcc01707*.

After cultures entered the stationary phase of phototrophic growth, the mutant SB1707 contained less BChl than the WT SB1003 strain, whereas SB1707(pCM1707) cells contained essentially the same amount as the WT strain (Figure 4B).

### Effects of Culture Growth Conditions on Coproporphyrin III-Accumulation

A recent study described a spontaneous *R. sphaeroides* mutant called PORF, which excretes a large amount of coprophyrin III and has a mutation in the *metK* gene that encodes S-adenosylmethionine (SAM) synthetase (Sabaty et al., 2010). It was proposed that depletion of SAM decreased the SAM-dependent coproporphyrinogen oxidase activity, leading to a coproporphyrin III-accumulation phenotype. To test whether a depletion of SAM caused coproporphyrin III accumulation by the mutant SB1707, 50 μM of SAM or 10 mM of the SAM precursor L-methionine were added to the minimal RCV-based medium with pyruvate as sole carbon source. It was found that neither addition of SAM nor L-methionine affected the amount of coproporphyrin III accumulated by SB1707 (data not shown).

It was observed that more coproporphyrin III was accumulated by mutant strain SB1707 when grown phototrophically in the complex medium YPS than in the minimal medium RCV, where the sole carbon source is malate (Supplementary Figure S4). Because *rcc01707* encodes an FBA predicted to function in central carbon metabolism, we investigated the effect of different carbon sources on coproporphyrin III accumulation by SB1707 and SB1003, including substances that require gluconeogenic activity (pyruvate, malate, succinate and serine), and sugars (glucose and fructose) that require glycolytic activity. The minimal medium RCV lacking malate was used as the base for addition of sole carbon sources. In initial experiments using either glucose or fructose as the sole carbon source, both SB1003 and SB1707 cultures had a pale color, and reached a relatively low optical density. The final pH of the media was found to be between 6 and 6.1, which is too acidic for *R. capsulatus* growth (Eidels and Preiss, 1970), and is probably due to the production of organic acids such as lactate, acetate and formate (Madigan et al., 1980). To avoid this problem we increased the buffering capacity of the media (called RCV/MOPS) containing sugars.

**FIGURE 5:**
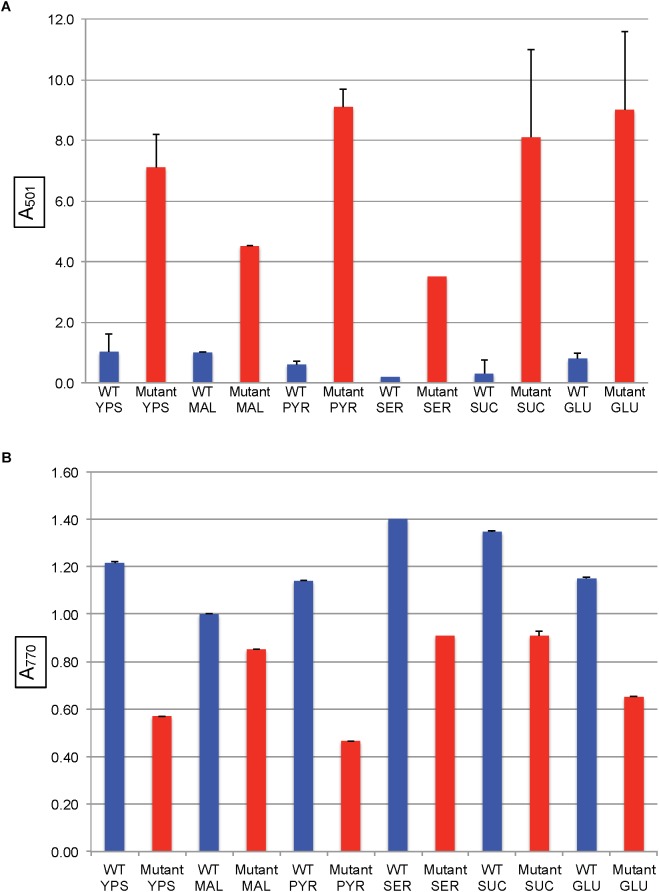
Pigment accumulation in cultures of the WT strain SB1003 and the mutant SB1707, grown phototrophically to the stationary phase in complex YPS medium, or in minimal media containing different sole sources of carbon as indicated: MAL, malate; PYR, pyruvate; SER, serine; SUC, succinate; GLU, glutamate. **(A)** Extracellular coproporphyrin III measured as A_501_ normalized to culture turbidity, shown on the vertical axis, relative to the WT strain SB1003 grown on malate. **(B)** Intracellular bacteriochlorophyll *a* measured as A_770_ normalized to culture turbidity, shown on the vertical axis, relative to the WT strain SB1003 grown on malate. Error bars give the range (*n* = 2) except for serine, which was done once.

In all of the modified RCV media with the above organic and amino acids as the sole carbon source, the SB1707 mutant accumulated more coproporphyrin III than the WT strain SB1003 (Figure 5A). Although there were differences between SB1707 cultures, none of the substances requiring gluconeogenic FBA activity yielded a clear correlation between the type of metabolic intermediate and the amount of coproporphyrin III accumulated. For example, the greatest amount of coproporphyrin III accumulation was observed with pyruvate as the sole carbon source, whereas the smallest amounts were found with serine or malate, which may be converted to pyruvate in one enzymatic step (Martinez-Luque et al., 2001). The TCA cycle intermediates malate and succinate differed from each other by about a factor of two, and glutamate (which is converted to succinate in three enzymatic steps) yielded the same amount of coproporphyrin III as succinate. Although the amounts of coproporphyrin III were very much lower in the WT strain, the effects of several of the carbon sources on coproporphyrin III accumulation on the WT strain SB1003 were qualitatively similar to SB1707 (Figure 5A and Supplementary Table S1). Therefore, the mutation in SB1707 seems to greatly exaggerate the effects of carbon sources on coproporphyrin III accumulation that exist in the WT strain. Cooper (1963) showed that a WT strain of *R. capsulatus* accumulates extracellular coproporphyrin III, which although it increases greatly under iron-deficient conditions is also present under iron-replete conditions.

The mutant strain SB1707 produced less BChl per cell than the WT strain, regardless of the carbon source (Figure 5B and Supplementary Table S2). In general, the greater the amount of coproporphyrin III accumulated the lesser the amount of BChl present, although there was not perfect correspondence.

**FIGURE 6:**
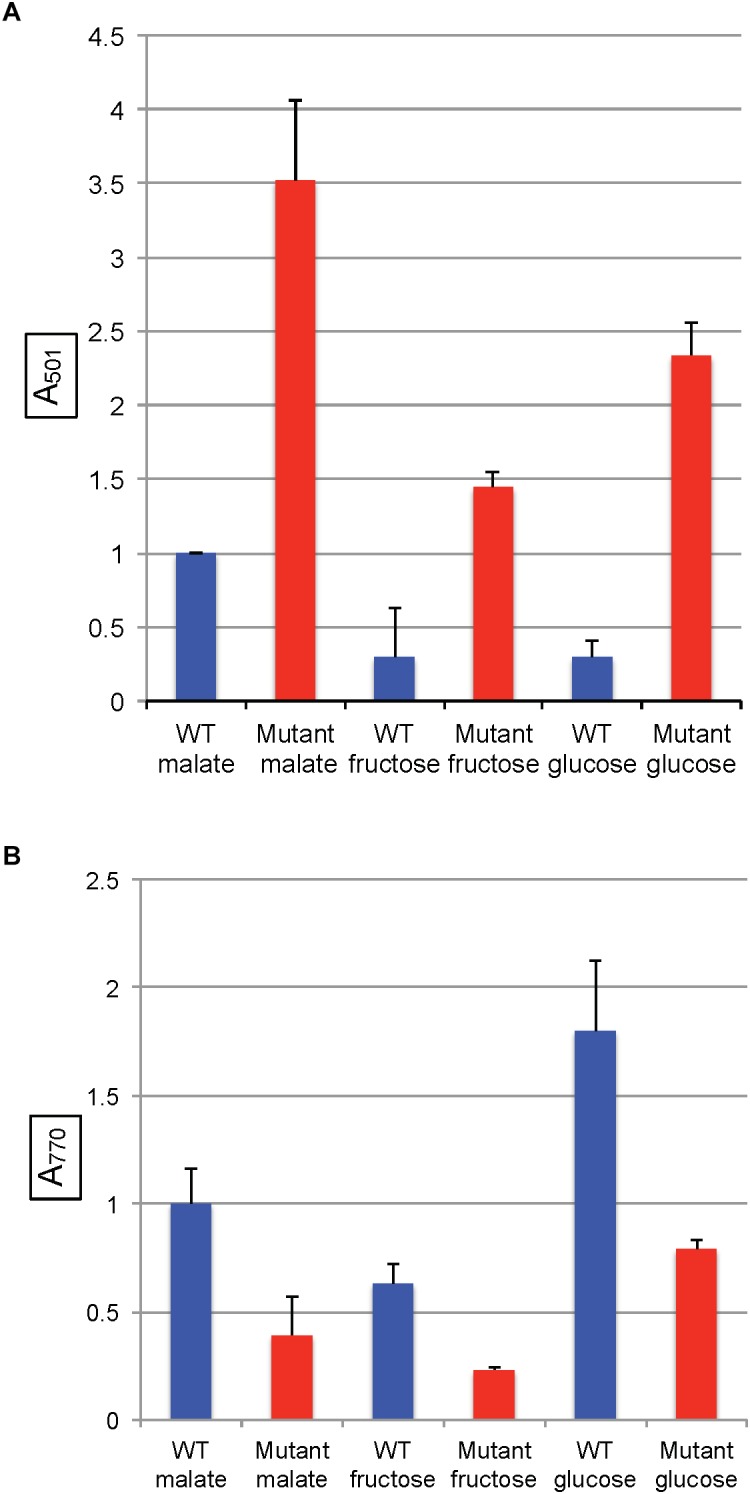
Pigment accumulation in cultures of the WT strain SB1003 and the mutant SB1707, grown phototrophically to the stationary phase in complex YPS medium, or in minimal media containing different sole sources of carbon as indicated. **(A)** Extracellular coproporphyrin III measured as A_501_ normalized to culture turbidity, shown on the vertical axis, relative to the WT strain SB1003 grown on malate. **(B)** Intracellular bacteriochlorophyll *a* measured as A_770_ normalized to culture turbidity, shown on the vertical axis, relative to the WT strain SB1003 grown on malate. Error bars give standard deviation, *n* = 3.

In *R. capsulatus*, glucose is converted to pyruvate through the Entner–Doudoroff pathway whereas the Embden-Meyerhof-Parnas (EMP) pathway is used for fructose (Conrad and Schlegel, 1977), and only the fructose pathway progresses through FBA to pyruvate (Figure 1). Although the absence of a glycolytic FBA might be thought to prevent growth on fructose, *R. capsulatus* is capable of converting fructose bisphosphate to fructose 6-phosphate (using fructose 1–6 diphosphatase), and thence to glucose 6-phosphate (using phosphoglucose isomerase), which then is catabolized to pyruvate through the FBA-independent Entner–Doudoroff pathway (Conrad and Schlegel, 1977). When either glucose or fructose was used as the sole carbon source, SB1707 excreted about 40–65% of the amount of coproporphyrin III as when malate was used as the sole carbon source in the same RCV/MOPS basal medium (Figure 6A). Again, as for the organic and amino acids, the differences seen with the mutant were qualitatively similar to the WT, which also accumulated less pigment when grown on sugars (Figure 6A and Supplementary Table S3).

As in the other media, the amounts of BChl synthesized in mutant cultures grown with sugars as the sole carbon source were less than in the WT controls (Figure 6B and Supplementary Table S4).

In all of the pairwise comparisons of the WT strain SB1003 to the mutant SB1707 the accumulation of coproporphyrin III was about 3.5 to 10-fold greater in mutant cultures, whereas the greatest difference in response of strain SB1707 to culture medium composition was about 2.5-fold. Although changes in the carbon source for growth had significant effects on the accumulation of coproporphyrin III, there was no clear indication of a possible metabolic bottleneck resulting from loss of FBA aldolase activity in either a glycolytic or a gluconeogenic pathway and the accumulation of coproporphyrin III.

### Expression of the Coproporphyrinogen III Oxidase Gene *hemN* Is Decreased in the Mutant Strain SB1707

**FIGURE 7:**
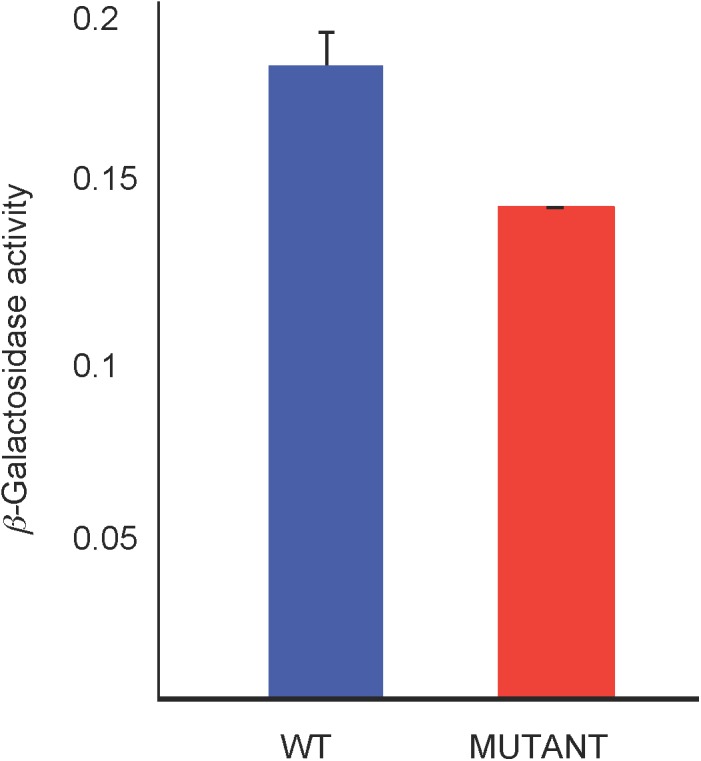
β-galactosidase activity from a plasmid-borne fusion of the *hemN* promoter and first seven codons in-frame with a *Escherichia coli lacZ* reporter in the WT strain SB1003 and mutant SB1707. Cells were from cultures grown phototrophically in RCV minimal medium. Error bars give the standard deviation, *n* = 3.

We used a *lacZ* reporter fused to the *hemN* promoter (in-frame with the *hemN* coding sequence) on plasmid pJS123 (Smart et al., 2004) to compare *hemN* expression levels in the WT strain SB1003 with the mutant SB1707. Cultures were grown phototrophically in the minimal medium RCV with malate as the sole carbon source. As shown in Figure 7, the amount of β-galactosidase activity in SB1707 cells was about 80% of that in SB1003 cells. Although this difference is modest, it is similar to the relative amounts of BChl in cells grown on this medium (Figure 5B), and consistent with a bottleneck rather than a block in the porphyrin biosynthetic pathway in the mutant strain SB1707.

**Table 1:** Bacterial strains and plasmids.

Bacteria	Relevant properties	Reference or source
*E. coli*		
DH5α λ-pir	*fhuA2 lac(del)U169 phoA glnV44 Φ80*′*lacZ(del)M15 gyrA96 recA1 relA1 endA1 thi-1 hsdR17,*λ-pir lysogen, allowing for replication of plasmids carrying oriR6K	Biomedal (Spain)
S17.1 λ-pir	Plasmid mobilizer strain with chromosome-integrated RP4 transfer genes.	Simon et al., 1983
		
*R. capsulatus*		
SB1003	Wild type; Rif^r^	Solioz and Marrs, 1977
SBpG	Chromosome-integrated GTA promoter driving fluorescent *mCherry* translational fusion	Kuchinski et al., 2016
SBT4-A13	Tn5pFru transposon mutant of SBpG with Tn5 inserted in *rcc01707*	This work
SB1707	*rcc01707::Tn5* GTA-transduced from SBT4-A13 into SB1003, Km^r^	This work
		
**Plasmids**		
pRL27	Hyperactive transposon Tn5 delivery plasmid	Larsen et al., 2002
pmCherry	Source of mCherry fluorescent protein gene	Clontech
pRLTn5pFru	Fructose promoter inserted into Tn5 in pRL27, facing out toward the transposon	This work
pJS123	*hemN* promoter and 5′-most seven codons fused in-frame to the *E. coli lacZ* gene	Smart et al., 2004

## Discussion

In a transposon mutagenesis screen for up-regulated mutants of a strain that produces the red fluorescent protein mCherry (Table 1), we identified the *R. capsulatus* mutant SBT4-A13, which excretes a large amount of red, fluorescent pigment into the culture medium. We found that this mutant has a disruption of *rcc01707*, encoding a putative Class I FBA. The RcGTA-mediated transduction of *rcc01707*::Tn5pfru from SBT4-A13 to the WT strain SB1003 resulted in the mutant strain SB1707 that also excretes a red pigment, and we identified the main compound accumulated in mutant cultures as coproporphyrin III. In addition, genetic complementation using the *rcc01707* WT allele confirmed that it is the mutation of the *rcc01707* gene that led to the coproporphyrin III accumulation phenotype.

Two types of porphyrin excretion mutants have been reported for *Rhodobacter* species. Some mutants excrete a combination of coproporphyrin III and protoporphytin IX, lack *c*-type cytochromes, and cannot grow phototrophically (Biel and Biel, 1990; Goldman et al., 1997; Deshmukh et al., 2000), and these mutants differ from the *R. capsulatus* mutant SB1707 described here, which is capable of phototrophic growth. Others, such as the *R. sphaeroides* “PORF” (*metK*) mutant (Sabaty et al., 2010) and the *R. gelatinosus copA* mutant (Azzouzi et al., 2013), resemble strain SB1707 because they are capable of phototrophic growth. However, the *rcc01707* gene differs from *metK*, which encodes S-adenosylmethionine (SAM) synthase, and addition of SAM or the SAM precursor methionine to the growth medium did not rescue the coproporphyrin III accumulation phenotype of SB1707. The *copA* mutant excretes pigment only in the presence of elevated levels of Cu^2+^, which again differs from the mutant SB1707.

The *R. capsulatus* gene *rcc01707* is annotated as a class I FBA, putatively catalyzing the glycolytic reaction that converts fructose 1, 6-bisphosphate to dihydroxy acetone phosphate and glyceraldehyde 3-phosphate. In the SB1003 genome, a class II FBA homolog (*rcc01830*) is also present. The two types of FBA are classified by the catalytic mechanism employed (Marsh and Lebherz, 1992), and these two FBAs encoded by *R. capsulatus* yield only 6% identity in a pairwise BLASTp. The *rcc01830* gene is located in an apparent operon with genes encoding CO_2_-fixation enzymes such as ribulose bisphosphate carboxylase, which indicates a gluconeogenic function for this FBA. Furthermore, it shares 75–95% sequence identity with two *R. sphaeroides* FBAs encoded in CO_2_ fixation operons (Chen et al., 1991; Gibson et al., 1991). In contrast, *rcc01707* is adjacent to a phosphoglycerate kinase and near pyruvate dehydrogenase genes, with nothing nearby to indicate a gluconeogenic function. In queries of the UniProtKB/Swiss-Prot and PDB databases, the top BLAST hits with known function were glycolytic Class I FBAs: the chemotrophic eubacterium *Porphyromonas gingivalis fda* gene product (PG_1755; E = e^−122^) and the cyanobacterium *Synechocystis* sp. PCC 6803 *slr0943* gene product (E = e^−118^) (Nakahara et al., 2003). Therefore it appears that the FBA encoded by *rcc01707* is a Class I enzyme that operates in glycolysis. This interpretation is supported by transcriptomic data obtained by Kumka et al. (2017), presented in their Supplementary Material. The transcripts of *rcc01707* did not change significantly between aerobic chemotrophic and anaerobic phototrophic growth (6,296 vs. 6,225 counts, respectively). In contrast, *rcc01830* transcripts increased 7.5-fold (2,249 to 16,797 counts) under phototrophic conditions, consistent with the well-established role of CO_2_ fixation and hence gluconeogenic FBA activity in maintaining redox homeostasis (Tichi and Tabita, 2001).

However, it has been found that some Class I and Class II FBAs may synthesize hexose in gluconeogenesis as well as degrade hexose in glycolysis (Scamuffa and Caprioli, 1980; Marsh and Lebherz, 1992). Because mutation of *rcc01707* does not abolish growth in the minimal medium RCV with fructose as the sole carbon source, it is possible that the Class II FBA encoded by *rcc01830* is capable of functioning in glycolysis as well as in its presumed normal gluconeogenic function. However, *R. capsulatus* is capable of converting fructose-bisphosphate to fructose 6-phosphate, and thence to glucose 6-phosphate (Figure 1), which is metabolized through an Entner–Doudoroff series of reactions to pyruvate (Conrad and Schlegel, 1977). Therefore there is no need to invoke a glycolytic activity for the Class II FBA encoded by *rcc01830*. When the mutant SB1707 was grown on a single organic or amino acid (such as pyruvate, malate, succinate, serine or glutamate) that is metabolically “downstream” of the FBA glycolytic reaction that converts fructose bisphosphate into dihydroxy and GAP, gluconeogenic activity must be present to provide appropriate amounts of sugars as precursors for essential metabolites. Evidently the *rcc01830* gene product provides this gluconeogenic activity in the mutant strain SB1707, as would be expected because of the genomic context of *rcc01830*, adjacent to CO_2_-fixation genes. Furthermore, if the loss of the FBA encoded by *rcc01707* resulted in a metabolic imbalance because of a bottleneck in gluconeogenesis, it would be expected that providing hexoses (glucose or fructose) would restore a near-WT phenotype. We found that during growth on either glucose or fructose the mutant cultures accumulated 4 to 7-fold more coproporphyrin III than the WT cultures grown in the same media (Figure 6A and Supplementary Table S3). Therefore the *rcc01707* mutation phenotype does not result from a deficiency in hexose.

If the loss of the FBA encoded by *rcc01707* resulted in a metabolic imbalance because of a bottleneck in glycolysis, it would be expected that providing pyruvate, TCA cycle intermediates, or amino acids that are readily converted to TCA cycle organic acids to the mutant would restore a near-WT phenotype. As shown in Figure 5A, we found that pyruvate-grown cultures of the mutant SB1707 accumulated > 10-fold more coproporphyrin III than the WT strain SB1003, and growth on TCA cycle intermediates and amino acids yielded ratios of mutant:WT accumulations ranging from 4.5 (malate) to 27 (succinate). Therefore the *rcc01707* mutation phenotype does not appear to result from a deficiency in pyruvate or TCA cycle intermediates.

We found that phototrophic growth of the mutant SB1707 is impaired when compared to the WT strain SB1003, because there was an extended lag phase for SB1707 to switch from aerobic dark to anaerobic phototrophic growth conditions, and the phototrophic growth of strain SB1707 was slower than that of the WT strain SB1003 (Supplementary Figure S3). We suggest that this impaired phototrophic growth is because there is a bottleneck in the synthesis of BChl required for phototrophic growth, at the step required for production of protoporphyrin IX, which explains the accumulation of coproporphyrinogen III. Indeed, regardless of carbon source, phototrophic cultures of the mutant SB1003 invariably contained less BChl than the WT strain SB1003 (Figure 4B, 5B).

Although our study did not reveal the exact mechanism leading to coproporphyrin III accumulation in the mutant strain SB1707, the β-galactosidase reporter data indicate that a decrease in *hemN* expression is at least partially responsible for a bottleneck in the porphyrin biosynthetic pathway. Other studies have shown that *hemN* expression is controlled by the transcription regulators RegA, FnrL, and HbrL (Smart et al., 2004; Smart and Bauer, 2006; Zappa and Bauer, 2013). As in some other coproporphyin III-accumulation mutants there is sufficient porphyrin synthesis in strain SB1707 to allow for the production BChl in reduced quantities, which support a slowed anaerobic phototrophic growth under the conditions used here.

It is possible that a metabolic imbalance causes the phenotype of the mutant strain SB1707, however, the continued high level of coproporphyrin III accumulation regardless of carbon source leads us to suggest an alternative possibility. In other organisms Class I and Class II FBAs may “moonlight” in non-aldolase activities, including the regulation of gene expression (Henderson and Martin, 2011; Ritterson Lew and Tolan, 2013; Shams et al., 2014; Ziveri et al., 2017; Snaebjornsson and Schulze, 2018). We speculate that the mutation of *rcc01707* results in the loss of a FBA moonlighting function needed for maximal expression of *hemN*, and a bottleneck in the conversion of coproporphyrinogen III to protoporphyrin IX.

## Materials and Methods

### Bacterial Strains and Plasmids, and Growth Conditions

The bacterial strains and plasmids used in this study are listed in Table 1. *R. capsulatus* strains were cultivated in either in YPS complex medium (Wall et al., 1975) or RCV minimal medium (Beatty and Gest, 1981b), except in some experiments where the 30 mM malate in the RCV medium was replaced by other carbon sources: glucose (13 mM), fructose (13 mM), pyruvate (40 mM), serine (40 mM), or succinate (24 mM). When a sugar was used as the sole source of carbon, the buffering capacity of the medium was increased by doubling the phosphate concentration from 9.6 to 19.2 mM, and addition of 3-(N-morpholino) propanesulfonic acid (MOPS; pH 7.0) at a concentration of 20 mM, called RCV/MOPS. The concentration of malate was 20 mM in identically buffered cultures used as controls for the cultures grown on sugars. When required, SAM (50 μM), or L-methionine (10 mM) were supplied in RCV medium. Anaerobic photoheterotrophic growth was carried out in 16.5-mL screw-cap tubes with tungsten filament lamp illumination of ∼150 μE m^−2^ s^−2^ (measured with a LI-COR quantum radiometer, model Li-185B, equipped with a PAR sensor). Antibiotics were included when required at concentrations of: kanamycin (10 μg/mL), tetracycline (1 μg/mL). *E. coli* strains were cultured aerobically at 37°C in LB medium supplemented with appropriate antibiotics at concentrations of: gentamicin (10 μg/mL), kanamycin (50 μg/mL), ampicillin (100 μg/mL), and tetracycline (10 μg/mL).

### Transposon Mutagenesis and Determination of Transposon Insertion Site

The *R. capsulatus* strain used in transposon mutagenesis, SBpG, was derived from the WT strain SB1003 by chromosomal insertion of an *mCherry* fluorescent protein gene fused to the gene transfer agent (RcGTA) major structural gene cluster promoter (Kuchinski et al., 2016). Plasmid pRL27 (Larsen et al., 2002) carrying the hyperactive Tn5 transposon was introduced into the *R. capsulatus* strain SBpG by conjugation using *E. coli* S17.1 (λ-pir) as the donor (Delorenzo and Timmis, 1994). Transposon recipients were selected on RCV agar containing kanamycin, and cells in colonies transferred to liquid RCV medium (containing kanamycin) in 384 well plates using a Qpix2 automatic colony picker (GENETIX). The cultures arising in the 384-well plates were screened for elevated fluorescence (excitation max: 590 nm; emission max: 610 nm) using a Varioskan Flash plate reader (Thermo Fisher Scientific). Coincidently, the fluorescence emission of coproporphyrin peaks at 610 nm, the same wavelength as that of the mCherry fluorescent protein.

To determine the Tn5 insertion site, genomic DNA of the transposon mutant was isolated, digested with either BamH I and/or Pst I, neither of which cut within the transposon. The digestion mixture was then re-ligated, and the resulting ligation mixture was used to transform competent DH5α λ-*pir* cells with selection of transformants on LB agar containing kanamycin. The location of the transposon in the *R. capsulatus* genome was determined by DNA-sequencing of plasmids isolated from transformants, using the Tn5 internal outward-facing primers Tn5pfruF (5′-CACTTTCTGGCTGGATGATG) and Tn5pfruR (5′-ATGAGCCTGTCGGCCTAC).

The disrupted gene was used to replace the WT allele of SB1003 by RcGTA-mediated gene transfer as previously described (Leung et al., 2012).

### Pigment Measurements

*Rhodobacter capsulatus* cultures were grown to the stationary phase, and cells were pelleted by centrifugation. The relative amounts of extracellular porphyrin were estimated by the A_501_ of the cell-free culture supernatant, normalized to culture turbidity on the basis of a Klett photometer reading (red filter #66; similar to the OD at 650 nm). The relative amounts of intracellular BChl were estimated by acetone extraction of cells obtained by centrifugation, and measurement of the A_770_, normalized to culture turbidity as described above. In some experiments the A_770_ of intact cells suspended in 60% sucrose was measured, again normalized to culture turbidity.

### HPLC Purification and Mass Spectral Analysis of Porphyrin Compounds

Culture supernatant was filtered through a 0.2 μm pore-diameter polyethersulfone membrane (VWR International), and directly injected into a 4.6 × 25 cm C18 HPLC column attached to a Waters, 2695 instrument fitted with a 200–800 nm photodiode array detector. Pigments were eluted from the column using a 0.8 ml/min flow rate with a linear gradient from 100% solvent A (18% methanol, 0.1 M ammonium acetate, pH 5.2) to 100% solvent B (90% methanol, 0.1 M ammonium acetate pH 5.2) in 3 min (Masuda et al., 1999). The retention time of each solute was monitored by the absorbance at 400 nm.

Mass spectrometry of HPLC-purified substances was performed at the University of British Columbia microanalysis and mass spectrometry facility using a MALDI-TOF mass spectrometer (Bruker). Briefly, the sample and DCTB matrix were dissolved in dichloromethane, and 1 μl was applied to the target and dried in air. The sample was run using the reflector mode and pulsed ion extraction. Analysis of results was aided by use of the online Isotope Distribution Calculator and Mass Spec Plotter (see footnote 1).

### β-Galactosidase Assay

Cultures were grown phototrophically in RCV minimal medium (Beatty and Gest, 1981b) to mid-late exponential phase (OD_650_ of 0.8–2, corresponding to ∼5 × 10^8^ – 10^9^ cfu/ml), harvested by centrifugation, and concentrated two-fold in Z-buffer for assay as based on Miller’s method (Miller, 1972). Cells (0.5 ml) were made permeable by addition of 50 μl each of chloroform and 0.1% sodium dodecyl sulfate, and vortexing. After 5 min at room temperature, 200 μl of *o*-nitrophenyl β-D-galactopyranoside were added, and after about 5 min the reaction was halted by the addition of 0.5 ml of 1 M Na_2_CO_3_. Cells were removed from the reaction mixture by centrifugation, and the absorbance of the supernatant was measured at 420 nm. The β-galactosidase activity is given here as the change in A_420_ divided by the incubation time in min and the OD_650_ of the cells suspended in Z buffer.

## Author Contributions

HD and JB designed the study, performed the experiments, made the figures, and wrote the manuscript. RS assisted in HPLC purification of the porphyrin compound. All authors edited the manuscript.

## Conflict of Interest Statement

The authors declare that the research was conducted in the absence of any commercial or financial relationships that could be construed as a potential conflict of interest.

## Footnotes

1 https://www.sisweb.com/mstools/isotope.htm

## References

[B1] Azzouzi A. Steunou A. S. Durand A. Khalfaoui-Hassani B. Bourbon M. L. Astier C. (2013). Coproporphyrin III excretion identifies the anaerobic coproporphyrinogen III oxidase HemN as a copper target in the Cu(+)-ATPase mutant copA(-) of *Rubrivivax gelatinosus*. *Mol. Microbiol.* 88 339–351. 10.1111/mmi.12188 23448658

[B2] Beatty J. T. Gest H. (1981a). Biosynthetic and bioenergetic functions of citric acid cycle reactions in *Rhodopseudomonas capsulata*. *J. Bacteriol.* 148 584–593. 7298578 10.1128/jb.148.2.584-593.1981 PMC216243

[B3] Beatty J. T. Gest H. (1981b). Generation of succinyl-coenzyme-A in photosynthetic bacteria. *Arch. Microbiol.* 129 335–340. 10.1007/BF00406457

[B4] Biel S. W. Biel A. J. (1990). Isolation of a *Rhodobacter capsulatus* mutant that lacks c-type cytochromes and excretes porphyrins. *J. Bacteriol.* 172 1321–1326. 10.1128/jb.172.3.1321-1326.1990 2155198 PMC208601

[B5] Chen J. H. Gibson J. L. Mccue L. A. Tabita F. R. (1991). Identification, expression, and deduced primary structure of transketolase and other enzymes encoded within the form-ii co2 fixation operon of *Rhodobacter sphaeroides*. *J. Biol. Chem.* 266 20447–20452. 1939098

[B6] Conrad R. Schlegel H. G. (1977). Different degradation pathways for glucose and fructose in *Rhodopseudomonas capsulata*. *Arch. Microbiol.* 112 39–48. 10.1007/BF00446652 139134

[B7] Cooper R. (1963). The Biosynthesis of coproporphyrinogen, magnesium protoporphyrin monomethyl ester and bacteriochlorophyll by *Rhodopseudomonas capsulata*. *Biochem. J.* 89 100–108. 10.1042/bj0890100 14097350 PMC1202277

[B8] Dailey H. A. Dailey T. A. Gerdes S. Jahn D. Jahn M. O’Brian M. R. (2017). Prokaryotic heme biosynthesis: multiple pathways to a common essential product. *Microbiol. Mol. Biol. Rev.* 81:e00048-16. 10.1128/MMBR.00048-16 28123057 PMC5312243

[B9] Daniels G. A. Drews G. Saier M. H. Jr. (1988). Properties of a Tn5 insertion mutant defective in the structural gene (fruA) of the fructose-specific phosphotransferase system of *Rhodobacter capsulatus* and cloning of the fru regulon. *J. Bacteriol.* 170 1698–1703. 10.1128/jb.170.4.1698-1703.1988 2832374 PMC211019

[B10] Delorenzo V. Timmis K. N. (1994). Analysis and construction of stable phenotypes in gram-negative bacteria with Tn5-derived and Tn10-derived minitransposons. *Methods Enzymol.* 235(Pt A), 386–405. 10.1016/0076-6879(94)35157-0 8057911

[B11] Deshmukh M. Brasseur G. Daldal F. (2000). Novel *Rhodobacter capsulatus* genes required for the biogenesis of various c-type cytochromes. *Mol. Microbiol.* 35 123–138. 10.1046/j.1365-2958.2000.01683.x 10632883

[B12] Eidels L. Preiss J. (1970). Carbohydrate metabolism in *Rhodopseudomonas capsulata*-enzyme titers, glucose metabolism, and polyglucose polymer synthesis. *Arch. Biochem. Biophys.* 140 75–89. 10.1016/0003-9861(70)90011-1 4248272

[B13] Gibson J. L. Falcone D. L. Tabita F. R. (1991). Nucleotide-sequence, transcriptional analysis, and expression of genes encoded within the form-I CO2 fixation operon of *Rhodobacter sphaeroides*. *J. Biol. Chem.* 266 14646–14653. 1907281

[B14] Goldman B. S. Beckman D. L. Bali A. Monika E. M. Gabbert K. K. Kranz R. G. (1997). Molecular and immunological analysis of an ABC transporter complex required for cytochrome c biogenesis. *J. Mol. Biol.* 268 724–738. 10.1006/jmbi.1997.0992 9175857

[B15] Gouda A. Fateen E. Nazim W. (2012). Porphyrins profile by high performance liquid chromatography/electrospray ionization tandem mass spectrometry for the diagnosis of porphyria. *J. Inherit. Metab. Dis.* 35:S161.

[B16] Henderson B. Martin A. (2011). Bacterial virulence in the moonlight: multitasking bacterial moonlighting proteins are virulence determinants in infectious disease. *Infect. Immun.* 79 3476–3491. 10.1128/IAI.00179-11 21646455 PMC3165470

[B17] Kuchinski K. S. Brimacombe C. A. Westbye A. B. Ding H. Beatty J. T. (2016). The SOS response master regulator LexA regulates the gene transfer agent of rhodobacter capsulatus and represses transcription of the signal transduction protein CckA. *J. Bacteriol.* 198 1137–1148. 10.1128/JB.00839-15 26833411 PMC4800879

[B18] Kumka J. E. Schindel H. Fang M. Zappa S. Bauer C. E. (2017). Transcriptomic analysis of aerobic respiratory and anaerobic photosynthetic states in Rhodobacter capsulatus and their modulation by global redox regulators RegA, FnrL and CrtJ. *Microb. Genom.* 3:e000125. 10.1099/mgen.0.000125 29114403 PMC5643017

[B19] LaMattina J. W. Nix D. B. Lanzilotta W. N. (2016). Radical new paradigm for heme degradation in *Escherichia coli* O157:H7. *Proc. Natl. Acad. Sci. U.S.A.* 113 12138–12143. 10.1073/pnas.1603209113 27791000 PMC5087033

[B20] Larsen R. A. Wilson M. M. Guss A. M. Metcalf W. W. (2002). Genetic analysis of pigment biosynthesis in *Xanthobacter autotrophicus* Py2 using a new, highly efficient transposon mutagenesis system that is functional in a wide variety of bacteria. *Arch. Microbiol.* 178 193–201. 10.1007/s00203-002-0442-2 12189420

[B21] Lascelles J. (1956). The synthesis of porphyrins and bacteriochlorophyll by cell suspensions of *Rhodopseudomonas spheroides*. *Biochem. J.* 62 78–93. 10.1042/bj0620078 13293156 PMC1274515

[B22] Lascelles J. (1966). The accumulation of bacteriochlorophyll precursors by mutant and wild-type strains of *Rhodopseudomonas spheroides*. *Biochem. J.* 100 175–183. 10.1042/bj1000175 5965250 PMC1265107

[B23] Leung M. M. Brimacombe C. A. Spiegelman G. B. Beatty J. T. (2012). The GtaR protein negatively regulates transcription of the gtaRI operon and modulates gene transfer agent (RcGTA) expression in *Rhodobacter capsulatus*. *Mol. Microbiol.* 83 759–774. 10.1111/j.1365-2958.2011.07963.x 22211723 PMC3641048

[B24] Madigan M. T. Cox J. C. Gest H. (1980). Physiology of dark fermentative growth of *Rhodopseudomonas capsulata*. *J. Bacteriol.* 142 908–915. 6769916 10.1128/jb.142.3.908-915.1980 PMC294116

[B25] Marsh J. J. Lebherz H. G. (1992). Fructose-bisphosphate aldolases: an evolutionary history. *Trends Biochem. Sci.* 17 110–113. 10.1016/0968-0004(92)90247-7 1412694

[B26] Martinez-Luque M. Castillo F. Blasco R. (2001). Assimilation of D-Malate by *Rhodobacter capsulatus* E1F1. *Curr. Microbiol.* 43 154–157. 10.1007/s002840010279 11400062

[B27] Masuda T. Inoue K. Masuda M. Nagayama M. Tamaki A. Ohta H. (1999). Magnesium insertion by magnesium chelatase in the biosynthesis of zinc bacteriochlorophyll a in an aerobic acidophilic bacterium *Acidiphilium rubrum*. *J. Biol. Chem.* 274 33594–33600. 10.1074/jbc.274.47.33594 10559247

[B28] Miller J. H. (1972). *Experiments in Molecular Genetics.* New York, NY: Cold Spring Harbor Laboratory.

[B29] Nakahara K. Yamamoto H. Miyake C. Yokota A. (2003). Purification and characterization of class-I and class-II fructose-1,6-bisphosphate aldolases from the cyanobacterium *Synechocystis* sp. *PCC* 6803. *Plant Cell Physiol.* 44 326–333. 12668779 10.1093/pcp/pcg044

[B30] Polo C. F. Frisardi A. L. Resnik E. R. Schoua A. E. Batlle A. M. (1988). Factors influencing fluorescence spectra of free porphyrins. *Clin. Chem.* 34 757–760. 3359615

[B31] Rebeiz C. (2002). “Analysis of intermediates and end products of the chlorophyll biosynthetic pathway,” in *Heme, Chlorophyll, and Bilins*, eds Smith A. Witty M. (New York, NY: Humana Press), 111–155.

[B32] Ritterson Lew C. Tolan D. R. (2013). Aldolase sequesters WASP and affects WASP/Arp2/3-stimulated actin dynamics. *J. Cell. Biochem.* 114 1928–1939. 10.1002/jcb.24538 23495010

[B33] Sabaty M. Adryanczyk G. Roustan C. Cuine S. Lamouroux C. Pignol D. (2010). Coproporphyrin excretion and low thiol levels caused by point mutation in the *Rhodobacter sphaeroides* S-adenosylmethionine synthetase gene. *J. Bacteriol.* 192 1238–1248. 10.1128/JB.01342-09 20038586 PMC2820862

[B34] Scamuffa M. D. Caprioli R. M. (1980). Comparison of the mechanisms of 2 distinct aldolases from *Escherichia coli* grown on gluconeogenic substrates. *BBA* 614 583–590. 10.1016/0005-2744(80)90247-8 6996735

[B35] Shams F. Oldfield N. J. Wooldridge K. G. Turner D. P. (2014). Fructose-1,6-bisphosphate aldolase (FBA)-a conserved glycolytic enzyme with virulence functions in bacteria: ‘ill met by moonlight’. *Biochem. Soc. Trans.* 42 1792–1795. 10.1042/BST20140203 25399608

[B36] Simon R. Priefer U. Puhler A. (1983). A broad host range mobilization system for in vivo genetic-engineering—transposon mutagenesis in gram-negative bacteria. *Nat. Biotechnol.* 1 784–791. 10.1038/nbt1183-784

[B37] Smart J. L. Bauer C. E. (2006). Tetrapyrrole biosynthesis in *Rhodobacter capsulatus* is transcriptionally regulated by the heme-binding regulatory protein, HbrL. *J. Bacteriol.* 188 1567–1576. 10.1128/JB.188.4.1567-1576.2006 16452440 PMC1367214

[B38] Smart J. L. Willett J. W. Bauer C. E. (2004). Regulation of *hem* gene expression in *Rhodobacter capsulatus* by redox and photosystem regulators RegA, CrtJ, FnrL, and AerR. *J. Mol. Biol.* 342 1171–1186. 10.1016/j.jmb.2004.08.007 15351643

[B39] Snaebjornsson M. T. Schulze A. (2018). Non-canonical functions of enzymes facilitate cross-talk between cell metabolic and regulatory pathways. *Exp. Mol. Med.* 50:34. 10.1038/s12276-018-0065-6 29657328 PMC5938058

[B40] Solioz M. Marrs B. (1977). The gene transfer agent of Rhodopseudomonas capsulata. *Purification and characterization of its nucleic acid*. *Arch. Biochem. Biophys.* 181 300–307. 10.1016/0003-9861(77)90508-2 879805

[B41] Tichi M. A. Tabita F. R. (2001). Interactive control of Rhodobacter capsulatus redox-balancing systems during phototrophic metabolism. *J. Bacteriol.* 183 6344–6354. 10.1128/JB.183.21.6344-6354.2001 11591679 PMC100130

[B42] Wall J. D. Weaver P. F. Gest H. (1975). Gene transfer agents, bacteriophages, and bacteriocins of *Rhodopseudomonas capsulata*. *Arch. Microbiol.* 105 217–224. 10.1007/BF00447140 1190956

[B43] Wu L. F. Saier M. H. Jr. (1990). Nucleotide sequence of the fruA gene, encoding the fructose permease of the Rhodobacter capsulatus phosphotransferase system, and analyses of the deduced protein sequence. *J. Bacteriol.* 172 7167–7178. 10.1128/jb.172.12.7167-7178.1990 2254279 PMC210842

[B44] Zappa S. Bauer C. E. (2013). The LysR-type transcription factor HbrL is a global regulator of iron homeostasis and porphyrin synthesis in Rhodobacter capsulatus. *Mol. Microbiol.* 90 1277–1292. 10.1111/mmi.12431 24134691 PMC3890261

[B45] Zappa S. Li K. R. Bauer C. E. (2010). The tetrapyrrole biosynthetic pathway and its regulation in *Rhodobacter capsulatus*. *Adv. Exp. Med. Biol.* 675 229–250. 10.1007/978-1-4419-1528-3_13 20532744 PMC2883787

[B46] Ziveri J. Tros F. Guerrera I. C. Chhuon C. Audry M. Dupuis M. (2017). The metabolic enzyme fructose-1,6-bisphosphate aldolase acts as a transcriptional regulator in pathogenic *Francisella*. *Nat. Commun.* 8:853. 10.1038/s41467-017-00889-7 29021545 PMC5636795

