# Supplementary material for: Porphyrin Excretion Resulting From Mutation of a Gene Encoding a Class I Fructose 1,6-Bisphosphate Aldolase in Rhodobacter capsulatus
Source: Front Microbiol. 2019 Feb 22;10:301. doi: 10.3389/fmicb.2019.00301 (PMC6395792; doi:10.3389/fmicb.2019.00301)
Supplement: Supplementary file 1 [file Data_Sheet_1.PDF]

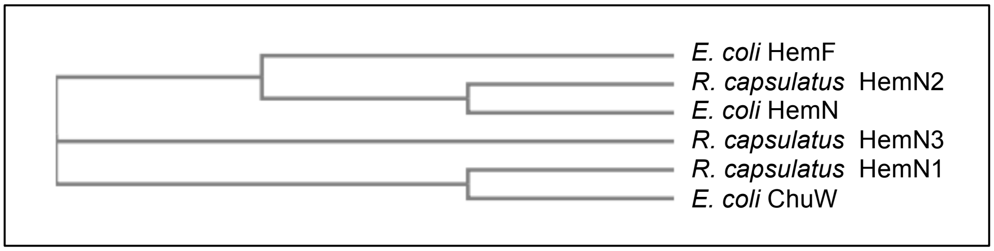

Figure S1. Neighbor-joining tree without distance corrections created using a MUSCLE alignment and ClustalW2 available at the https://www.ebi.ac.uk/Tools/msa/ web site.

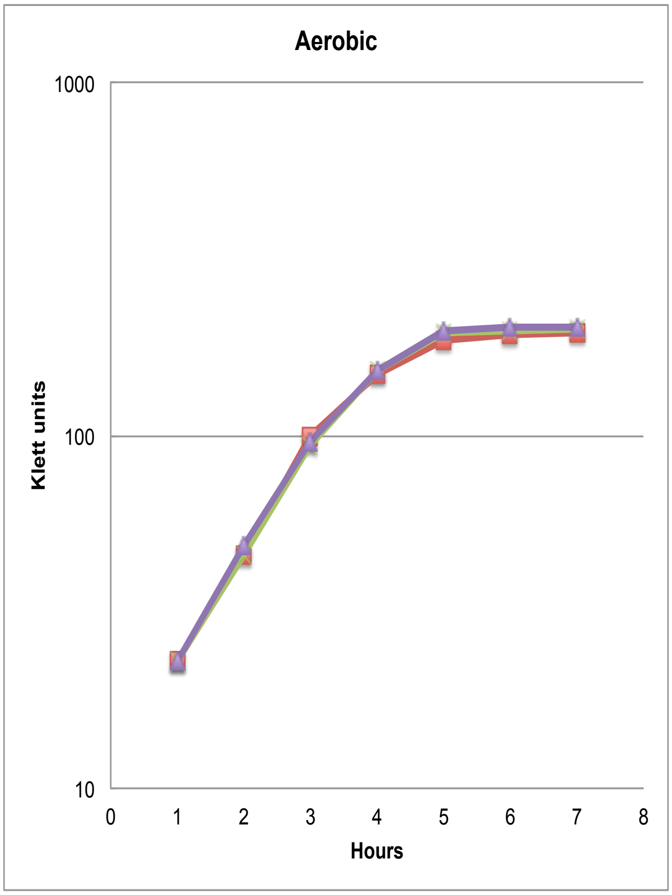

Figure S2. Chemotrophic (aerobic, not illuminated) growth kinetics in liquid complex medium YPS. The WT strain SB1003 is represented by squares, the mutant strain SB1707 by circles, and strain SB1707 complemented in *trans* (plasmid pCM1707) by triangles.

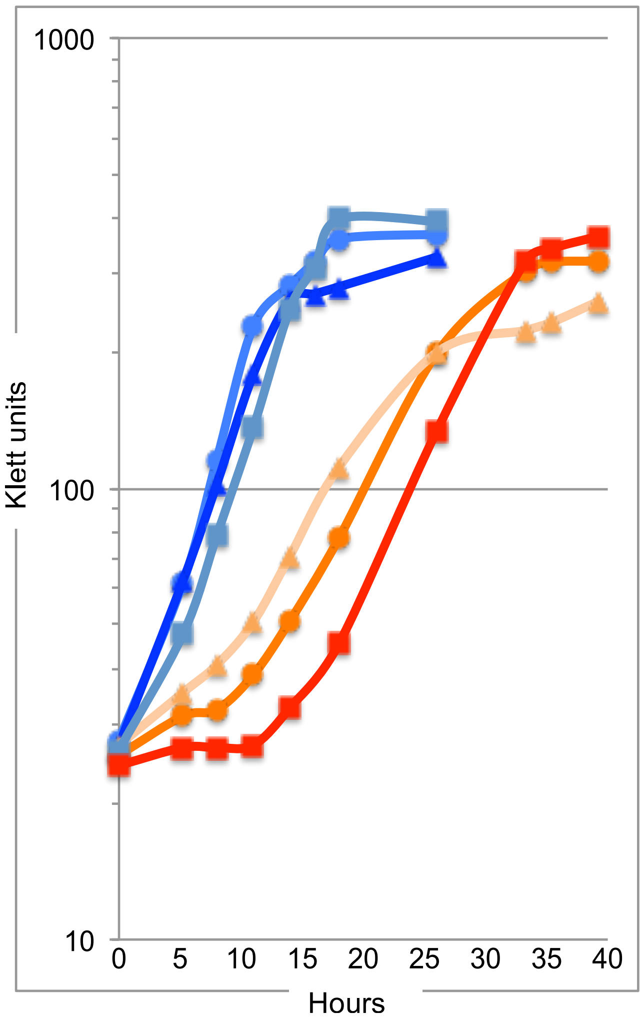

Figure S3. Phototrophic growth kinetics in liquid YPS complex medium. Inocula were grown chemotrophically (as in Figure S1), and transferred to screw-cap sealed tubes, which were illuminated to promote growth. The shades of blue represent the WT strain SB1003 (exponential phase doubling times ~ 3 h), and the shades of orange represent the mutant SB1707 (exponential phase doubling times ~ 6 h). Cultures grown on malate as the sole carbon source are represented by circles; fructose-grown cultures are indicated by triangles; glucose-grown cultures are indicated by squares.

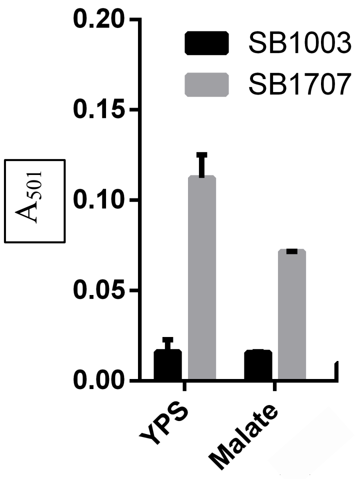

Figure S4. Pigment accumulation in cultures of the WT strain SB1003 and the mutant SB1707, grown phototrophically to the stationary phase in complex YPS or in minimal RCV medium containing malate as the sole source of carbon. Extracellular coproporphyrin III measured as A_{501} normalized to culture turbidity, shown on the vertical axis. Error bars give the range of values, n = 2.

Table S1. Data for Figure 5A. Extracellular coproporphyrin III measured as A_{501} normalized to culture turbidity.

WT YPS	Mutant YPS	WT MAL	Mutant MAL	WT PYR	Mutant PYR	WT SER	Mutant SER	WT SUC	Mutant SUC	WT GLU	Mutant GLU
0.016	0.112	0.016	0.072	0.010	0.144	0.003	0.055	0.005	0.128	0.012	0.142

Table S2. Data for Figure 5B. Intracellular BChl *a* measured as A_{770} normalized to culture turbidity.

WT YPS	Mutant YPS	WT MAL	Mutant MAL	WT PYR	Mutant PYR	WT SER	Mutant SER	WT SUC	Mutant SUC	WT GLU	Mutant GLU
0.524	0.246	0.431	0.367	0.492	0.200	0.604	0.392	0.581	0.392	0.496	0.280

Table S3. Data for Figure 6A. Extracellular coproporphyrin III measured as A_{501} normalized to culture turbidity.

WT malate	Mutant malate	WT fructose	Mutant fructose	WT glucose	Mutant glucose
0.009	0.032	0.003	0.013	0.003	0.021

Table S4. Data for Figure 6B. Intracellular BChl *a* measured as A_{770} normalized to culture turbidity.

WT malate	Mutant malate	WT fructose	Mutant fructose	WT glucose	Mutant glucose
0.295	0.114	0.186	0.069	0.544	0.233
